# Gene and transcript abundances of bacterial type III secretion systems from the rumen microbiome are correlated with methane yield in sheep

**DOI:** 10.1186/s13104-017-2671-0

**Published:** 2017-08-08

**Authors:** Janine Kamke, Priya Soni, Yang Li, Siva Ganesh, William J. Kelly, Sinead C. Leahy, Weibing Shi, Jeff Froula, Edward M. Rubin, Graeme T. Attwood

**Affiliations:** 1 AgResearch Limited, Grasslands Research Centre, Tennent Drive, Palmerston North, 4442 New Zealand; 2 Department of Energy, Joint Genome Institute, Walnut Creek, CA 94598 USA; 3 Genomic Division, Lawrence Berkeley National Laboratory, Berkeley, CA 94720 USA

**Keywords:** Rumen, Methane, Bacterial, Type III secretion, *Succinivibrio*

## Abstract

**Background:**

Ruminants are important contributors to global methane emissions via microbial fermentation in their reticulo-rumens. This study is part of a larger program, characterising the rumen microbiomes of sheep which vary naturally in methane yield (g CH_4_/kg DM/day) and aims to define differences in microbial communities, and in gene and transcript abundances that can explain the animal methane phenotype.

**Methods:**

Rumen microbiome metagenomic and metatranscriptomic data were analysed by Gene Set Enrichment, sparse partial least squares regression and the Wilcoxon Rank Sum test to estimate correlations between specific KEGG bacterial pathways/genes and high methane yield in sheep. KEGG genes enriched in high methane yield sheep were reassembled from raw reads and existing contigs and analysed by MEGAN to predict their phylogenetic origin. Protein coding sequences from *Succinivibrio dextrinosolvens* strains were analysed using Effective DB to predict bacterial type III secreted proteins. The effect of *S. dextrinosolvens* strain H5 growth on methane formation by rumen methanogens was explored using co-cultures.

**Results:**

Detailed analysis of the rumen microbiomes of high methane yield sheep shows that gene and transcript abundances of bacterial type III secretion system genes are positively correlated with methane yield in sheep. Most of the bacterial type III secretion system genes could not be assigned to a particular bacterial group, but several genes were affiliated with the genus *Succinivibrio,* and searches of bacterial genome sequences found that strains of *S. dextrinosolvens* were part of a small group of rumen bacteria that encode this type of secretion system. In co-culture experiments, *S*. *dextrinosolvens* strain H5 showed a growth-enhancing effect on a methanogen belonging to the order Methanomassiliicoccales, and inhibition of a representative of the *Methanobrevibacter gottschalkii* clade.

**Conclusions:**

This is the first report of bacterial type III secretion system genes being associated with high methane emissions in ruminants, and identifies these secretions systems as potential new targets for methane mitigation research. The effects of *S. dextrinosolvens* on the growth of rumen methanogens in co-cultures indicate that bacteria-methanogen interactions are important modulators of methane production in ruminant animals.

**Electronic supplementary material:**

The online version of this article (doi:10.1186/s13104-017-2671-0) contains supplementary material, which is available to authorized users.

## Background

Methane emissions from enteric fermentation in livestock are important contributors to global warming [1, 2] and most of these emissions are from the action of methanogenic archaea in the reticulo-rumen of ruminant animals. In the reticulo-rumen, methanogens use the products of bacterial fermentation, (hydrogen, carbon dioxide, formate and methyl compounds) to produce methane [3, 4]. Considerable amounts of methane are produced each day, a typical sheep produces ~40 L of methane per day, while a dairy cow belches around 450 L each day [5–7]. Methane is a particularly strong greenhouse gas with a global warming potential of 34× that of CO_2_ [1], and many researchers around the world are investigating ways to reduce methane emissions from ruminants. Animal selection is being investigated as a means of breeding low methane emitting animals [8–14], and sheep with consistently high methane yields (HMY; defined in g methane/kg dry matter intake DMI/day) or low methane yield (LMY) have been identified. The methane yield trait is heritable [8], and the animal characteristics controlling feed particle retention time [15–18] and rumen volume [10] are likely to contribute to the trait. Microorganisms in the rumen also contribute to the methane yield phenotype, and differences between HMY and LMY sheep in rumen microbiome composition [19] and methanogen gene expression [20] have been reported. We have recently found that methanogen changes in LMY animals are paralleled by an enrichment in *Sharpea* spp. in the bacterial community and higher abundance of the genes and transcripts encoding lactate formation and metabolism pathways that lead to lower hydrogen production and therefore lower methane formation in the rumen [21]. Here, we report the analysis of the rumen microbiomes of HMY animals and report the finding that gene and transcript abundances of bacterial type III secretion system (T3SS) genes are positively correlated with HMY in sheep.

## Methods

### Study aim and design

This report is part of a larger study [20], the aim of which was to characterise the rumen microbiomes of sheep which differed in their methane yield (g CH_4_/kg DMI/day) to investigate whether there were corresponding differences in microbial communities, and in gene and transcript abundances that could explain the animal methane phenotype. Rams (11 HMY and 11 LMY) were selected based on their methane yields and breeding values [8] and their methane yields were re-measured twice (measurements were separated by 2 weeks) in respiration chambers at the New Zealand Ruminant Methane Measurement Centre, AgResearch Grasslands, Palmerston North, New Zealand, on a pelleted lucerne diet [20]. Samples of rumen contents were collected at the end of each methane measurement period, by stomach intubation 4 h after the morning feeding. The pH of rumen contents was measured and the samples were immediately snap-frozen as pellets in liquid N_2_, and stored at −85 °C prior to DNA and RNA extractions. Based on the re-measured methane yields, frozen rumen samples were selected from 4 HMY (group mean 15.85 g CH_4_/kg DMI/day) and 4 LMY (group mean 11.44 g CH_4_/kg DMI/day) animals (one sample each per methane measurement, 16 samples), and from 2 animals which had intermediate methane yield (IMY, group mean 13.77 g CH_4_/kg DMI/day) (one each at the two methane measurement points, 4 samples) and used for DNA and RNA extractions (20 samples for each extraction). Nucleic acids extractions, purifications and library construction followed protocols described in the parent study [20]. Briefly, DNA was extracted using the “Repeated Bead Beating and Column (RBB + C) purification” method [22]. For large paired-end insert libraries, high-molecular-weight DNA was extracted using the method of Rosewarne et al. [23]. RNA extraction was via a hot lysis-acid phenol extraction method [20]. For cDNA library construction, total RNA was enriched for mRNA using the Ribo-Zero TM rRNA Removal Kit (Meta-Bacteria, Epicenter Biotechnologies, Madison, WI, USA), and fragmented using mRNA Fragmentation Reagents (Ambion, Foster City, CA, USA). Double-stranded cDNA (ds cDNA) was synthesised using SuperScript II reverse transcriptase (Invitrogen, Carlsbad, CA, USA), using random hexamers (MBI Fermentas, NY, USA). The cDNA sequencing libraries were generated and amplified using the Illumina TruSeqTM genomic sample prep kit (Illumina, San Diego, CA, USA) following the manufacturer’s instructions. The amplified libraries were purified and size-selected and the pooled library was sequenced using the Illumina HiSeq 2000 platform. The metagenomic sequencing produced, on average, 216 M reads per sample (51 Gb sequence), while the metatranscriptome sequencing produced 35 M reads (5.3 Gb non-rRNA sequence) per sample. All sequencing data used in this study has been previously published by our group and detailed description of data processing can be found in these publications [20, 21]. Briefly, quality-checked 16S rRNA amplicon sequencing data was processed using the QIME pipeline [24] and phylogenetically assigned using the Greengenes database (version gg_13_5) [25]. Metagenomic and metatranscriptome sequencing datasets were quality and artifact filtered and paired end reads merged using FLASH [26] and used for read count based comparative analysis by screening against the KEGG database [27], (version 58.1) using USEARCH 6.0 [28] at an E-value cutoff of 1 × 10^−5^ and read count matrices were constructed and normalised to reads per million (RPM).

Analysis of fermentation acids from rumen samples was performed on a Shimadzu 5050a GC–MS (Shimadzu, Kyoto, Japan) equipped with a ZB-5 MS (30 m × 0.25 mm ID × 0.25 µm film thickness, Phenomenex, USA) capillary column. Acids were extracted from the acidified samples with ether and derivatised to their *t*-butyl-dimethylsilyl esters, which detects both volatile and non-volatile fatty acids.

### Data analyses

Statistical analyses were conducted using reads per million (RPM)-normalised read count matrices as previously described [21]. Gene set enrichment analysis (GSEA, [29]) was used to estimate differential gene and transcript abundances between HMY or LMY animals (n = 16) at the pathway level, based on KEGG pathways. The GSEA-P application [29] was used to pre-rank genes based on signal-to-noise metric scores and to estimate normalised enrichment scores (NES), nominal *P* values and false discovery rates (FDR) with 10,000 phenotype permutations. Sparse partial least squares regression analysis (sPLS) in R [30] was used to estimate correlations between specific KEGG genes and methane yield, using the spls package [31]. Genes selected as methane predictors were assessed using the optimum sparsity tuning parameter (eta) and number of hidden components (K) predicted in the mean squared prediction error plot (MSPE). KEGG gene predictors with confidence intervals (95%) shifting from positive to negative correlation or from negative to positive, were excluded from the predictor gene set. The selected KEGG gene sets of the sPLS regression analysis of each dataset were manually compared with the results of categorical statistical analysis using Wilcoxon Rank Sum (WRS) test (10,000 permutations) [20] and GSEA pathways enrichment scores. For all statistical analyses a significance threshold of Benjamini Hochberg (BH) corrected *P* < 0.05 was used. Subsequent analyses concentrated on KEGG gene sets or gene categories supported by two or more methods of analysis.

### Read extraction and assembly

To gain an overview of the taxonomic origin of bacterial T3SS genes we reassembled these genes based on raw reads and contigs from existing assemblies, with hits to any of the KEGG genes encoding for the relevant subunits (K03219, K03221–K03230, K04056–K04059) from both metagenome and metatranscriptome data. Individual assemblies based on the 20 metagenome and 20 metatranscriptome datasets were constructed as previously described [20]. Assemblies were combined, and duplicated contigs, or smaller contigs covered by larger ones, were removed using the clustering function in Vmatch (http://www.vmatch.de). The resulting contigs were submitted to IMG/Mer [32] for gene-calling and automatic annotation. All assembled genes with hits to the relevant KEGG genes were extracted and combined with the corresponding reads from metagenome and metatranscriptome data. Contigs were extended using the Distributed Nucleating Assembler function in Kmernator (https://github.com/JGI-Bioinformatics/Kmernator). Genes on the resulting contigs were predicted using MetaGeneMark [33].

### Phylogenetic assignments of bacterial T3SS genes and prediction of effector proteins

Re-assembled contigs that contained partial and full length T3SS genes were imported into Geneious v.7.0.5 (Biomatters Ltd, Auckland, New Zealand) and de novo assembled into larger contigs where possible. The resulting 73 contigs were BLAST searched against the NCBI non-redundant database using BLASTX [34]. The output was analysed using MEGAN5 [35] for phylogenetic assignment using default settings. Matches were confirmed by homology searches of relevant genes in the IMG/M database [32]. Protein coding sequences from the draft genomes of *S. dextrinosolvens* strains H5, 22B and ACV-10 were analysed using EffectiveDB [36], which uses various software tools to predict bacterial secreted proteins based on their amino acid sequences. These tools include EffectiveT3 for prediction of Type III secretion signals, EffectiveCCBD for detection of conserved binding domains of Type III chaperones, EffectiveELD for secretion system independent prediction of secreted proteins based on eukaryotic-like domains, T4SEpre for recognition of Type IV secreted proteins and Predotar to screen N-terminal targeting sequences to predict their subcellular localization in eukaryotic host cells.

### Co-culture experiments

Cultures of rumen methanogens were inoculated with *Succinivibrio dextrinosolvens* strain H5 to explore its effects on methane production in vitro. Two *Methanobrevibacter* species were used, representing hydrogenotrophic methanogens within the *Mbb. ruminantium* clade (*Mbb. ruminantium* M1) or *Mbb. gottschalkii* clade (*Mbb. millerae* SM9), along with two methylotrophs representing the *Methanosphaera* clade (*Methanosphaera* sp. ISO3-F5) and the order Methanomassiliicoccales, (methanogenic archaeon ISO4-H5). Each methanogen was grown with *S.* *dextrinosolvens* strain H5 in either RM02 medium [37] with hydrogen + carbon dioxide (for the hydrogenotrophic methanogens, 1 atm over-pressure of a 80:20, hydrogen:carbon dioxide) or BY medium [38] with methanol + hydrogen (1 atm over pressure of 80:20, hydrogen:carbon dioxide + 20 mM methanol). Both media contained acetate (20 mM) and coenzyme M (1 mM). The hydrogenotrophic and methylotrophic methanogen cultures were grown in their respective media until methane was first detected in the culture headspace, then were inoculated with 10% inoculum *S.* *dextrinosolvens* strain H5 with glucose (10 mM final concentration) or pectin (1% pectin final concentration) added as a substrate for the *S. dextrinosolvens*, respectively. Incubation was continued until maximal methane production was detected in the control tubes. Control tubes contained methanogen cultures which received only the growth substrate without the *S. dextrinosolvens* inoculum. The pHs of the cultures were measured after inoculation and after completion of growth using pH indicator strips (Merck KGaA, Darmstadt, Germany). Methane and hydrogen concentrations in the culture headspaces were determined by removing a sample of the gases and analysing by gas chromatography (Aerograph 660, Wilkins Instruments & Research Inc., Walnut Creek, CA, USA) against methane and hydrogen standards. Samples of cultures were taken before and after incubation for analysis by gas–liquid chromatography to determine production or use of volatile fatty acids. Samples were centrifuged (21,000×*g* at 4 °C for 10 min) and 0.9 mL of the supernatant was added to 0.1 mL of internal standard solution (19 mM ethyl butyrate in 20% (v/v) phosphoric acid). Samples were kept at −20 °C until analysis, when they were thawed, clarified by centrifugation (21,000×*g* at 4 °C for 10 min) and 0.8 mL of the supernatant was transferred into a 2-mL crimp cap vial for analysis by gas–liquid chromatography. Supernatant samples were analysed on a nitroterephthalic acid-modified polyethylene glycol column (DB-FFAP; 30 m × 0.53 mm × 1.0 µm film thickness; J & W Scientific, Folsom, CA, USA) attached to a Hewlett-Packard 6890 series gas chromatography system, using helium as the carrier gas (5 mL/min). The oven temperature started at 85 °C, ramped to 200 °C at 10 °C/min, was held at 200 °C for 10 min, and then decreased to 50 °C and held for 5 min before the next sample was injected. Peaks were detected with a flame ionization detector, identified by comparison with standards, and integrated with Hewlett-Packard ChemStation software (version 4.02).

### Animal ethics approval

The collections of rumen contents from sheep were carried out under the approval of the AgResearch Ltd Grasslands Animal Ethics Committee (Approval 13606).

### Consent to participate and publish

Not applicable.

## Results

### T3SS genes and transcripts are enriched in HMY animals

**Fig. 1:**
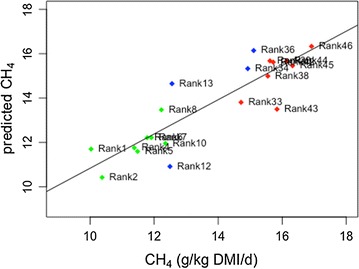
Multiple regression plot of 10 bacterial T3SS subunit genes. The multiple regression plot is based on partial least squares prediction of correlation coefficient over all 20 samples. Low methane yield animals are shown in *green*, intermediate methane yield animals in *blue* and high methane yield animals in *red*. Adjusted multiple regression coefficient R^2^ = 0.76, *P* = 3.48 × 10^−7^

**Fig. 2:**
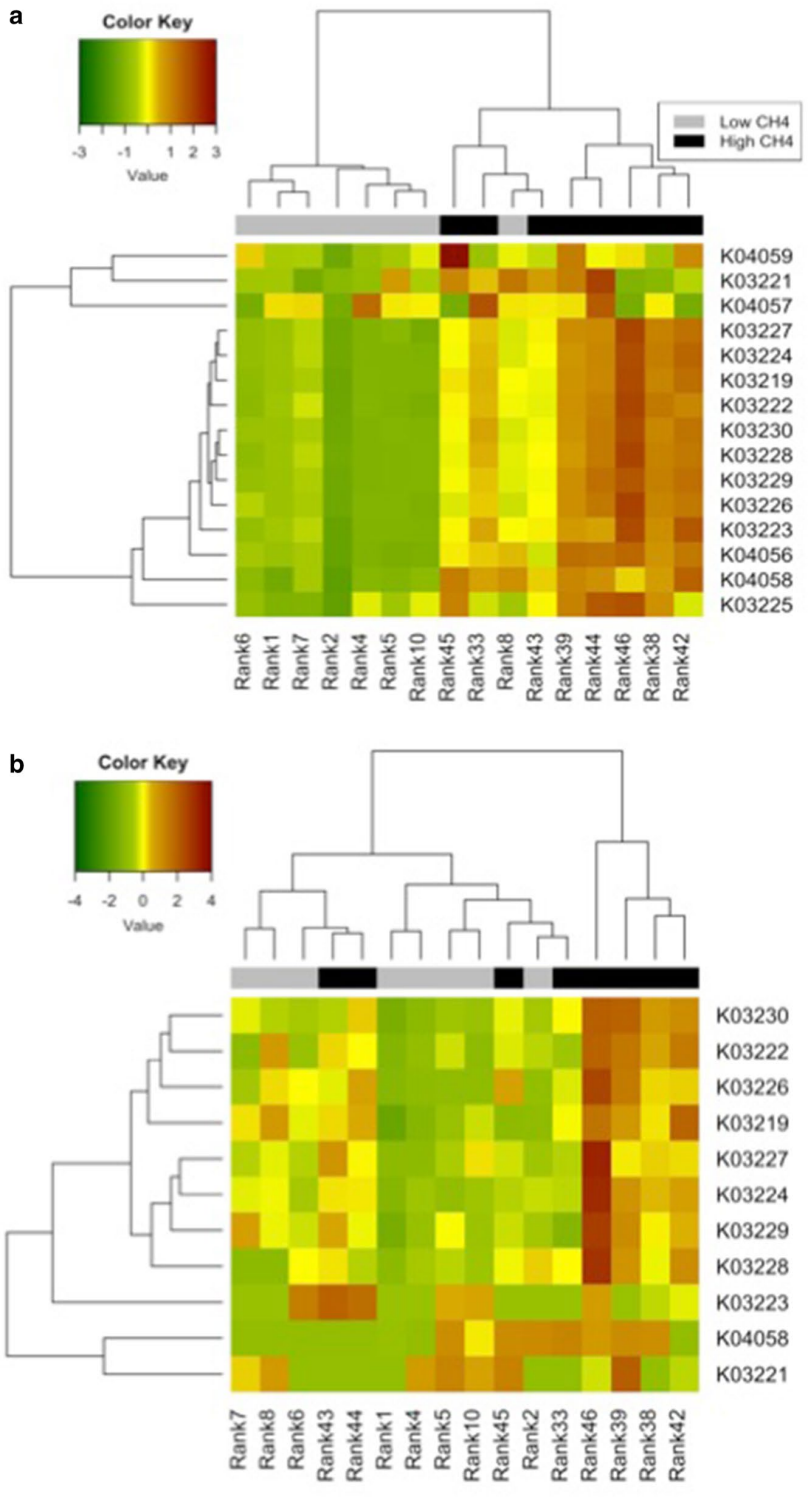
Z-score based hierarchical clustering of bacterial T3SS genes. Hierarchical clustering based on metagenome (**a**) and metatranscriptome (**b**) data

The metagenomic and metatranscriptomic data generated from rumen samples collected from LMY and HMY sheep were analysed by sPLS and the WRS test, and 60 genes and 36 transcripts were significantly (BH corrected *P* < 0.05) more abundant in the HMY animals in both analyses (Additional file 1). Those genes and transcripts that were twofold or greater more abundant in the HMY animals were enriched for KEGG genes related to bacterial T3SS subunits and are listed in Table 1. In the WRS test, 12 out of a total of 15 subunits known to be involved in bacterial T3SSs had significantly more reads in the HMY animals. Ten T3SS subunits were also identified as correlation predictors in the sPLS analysis (Table 2) with an adjusted multiple regression coefficient of R^2^ = 0.76, (*P* = 3.48 × 10^−7^, Fig. 1). At the transcriptome level, a similar but weaker trend was observed, with two subunits showing significantly higher transcript abundance in HMY animals and one subunit being selected as a predictor gene for methane yield in the sPLS regression analysis (Table 2). The GSEA identified Bacterial secretion systems (ko03070) as the top ranked pathway represented in the metagenome dataset associated with HMY animals (Table 3) along with Drug metabolism and other enzymes (ko00983) and Alanine, aspartate and glutamate metabolism (ko00250) which showed a similar trend. Hierarchical clustering analysis based on Z-scores for both metagenome and metatranscriptome data, provided further support for differential T3SS gene abundance and expression between HMY and LMY animals (Fig. 2).

**Table 1:** T3SS genes significantly enriched in metagenome and metatranscriptome datasets from HMY animals

Dataset	KEGG gene	Definition/gene name	Mean RPM		WRS		sPLS coefficient
			LMY ± SD	HMY ± SD	Fold change	P adj. BH	
Metagenome	K03224	ATP synthase type III secretion protein *sctN*	2.47 ± 1.22	8.32 ± 2.45	3.37	<0.01	0.02
	K03227	Type III secretion protein *sctS*	0.46 ± 0.23	1.54 ± 0.44	3.33	<0.01	0.02
	K03230	Type III secretion protein *sctV*	3.68 ± 1.64	12.2 ± 3.52	3.32	<0.01	0.02
	K03228	Type III secretion protein *sctT*	1.11 ± 0.56	3.57 ± 1.10	3.21	<0.01	0.02
	K03229	Type III secretion protein *sctU*	1.60 ± 0.72	5.05 ± 1.47	3.16	<0.01	0.02
	K03226	Type III secretion protein *sctR*	1.23 ± 0.52	3.78 ± 1.21	3.06	<0.01	0.01
	K03223	Type III secretion protein *sctL*	0.46 ± 0.23	1.31 ± 0.38	2.83	<0.01	0.02
	K03222	Type III secretion protein *sctJ*	0.81 ± 0.44	2.52 ± 0.74	3.13	0.02	0.02
	K03219	Type III secretion protein *sctC*	1.22 ± 0.60	3.71 ± 1.03	3.03	0.02	0.02
	K13853	3-deoxy-7-phosphoheptulonate synthase/chorismate mutase *aroG, aroA*	0.14 ± 0.04	0.38 ± 0.39	2.82	0.03	NA
	K04058	Type III secretion protein *sctW*	0.17 ± 0.10	0.40 ± 0.07	2.33	0.05	0.02
	K04056	Type III secretion protein *sctO*	0.11 ± 0.07	0.30 ± 0.08	2.72	0.05	0.02
Metatranscriptome	K03230	Type III secretion protein *sctV*	0.98 ± 0.33	2.42 ± 0.97	2.47	0.03	NA
	K00814	Alanine transaminase *GPT*	2.15 ± 0.77	4.77 ± 1.25	2.21	0.03	0.03
	K00772	5′-methylthioadenosine phosphorylase *mtaP*	3.07 ± 0.84	7.64 ± 5.98	2.49	0.04	NA
	K03226	Type III secretion protein *sctR*	0.18 ± 0.15	0.62 ± 0.30	3.41	0.04	0.03

**Table 2:** T3SS subunits identified as correlation predictors in sPLS analysis

T3SS protein	KEGG	LMY	HMY	*P*	*P* adj. BH	Fold change	sPLS coefficient	Sample type
SctC	K03219	1.22	3.71	0.00	0.02	3.03	0.02	DNA
SctF	K03221	0.06	0.05	0.92	1.00	1.22	na	DNA
SctJ	K03222	0.80	2.52	0.00	0.02	3.13	0.02	DNA
SctL	K03223	0.46	1.31	0.00	0.01	2.83	0.02	DNA
SctN ATP synthase	K03224	2.47	8.32	0.00	0.01	3.37	0.02	DNA
SctQ	K03225	0.04	0.07	0.00	0.05	1.95	0.02	DNA
SctR	K03226	1.23	3.78	0.00	0.01	3.06	0.01	DNA
SctS	K03227	0.46	1.54	0.00	0.01	3.33	0.02	DNA
SctT	K03228	1.11	3.57	0.00	0.01	3.21	0.01	DNA
SctU	K03229	1.60	5.05	0.00	0.01	3.16	0.02	DNA
SctV	K03230	3.68	12.22	0.00	0.01	3.32	0.02	DNA
SctO	K04056	0.11	0.30	0.00	0.05	2.72	0.02	DNA
SctP	K04057	0.01	0.00	0.65	1.00	1.23	na	DNA
SctW	K04058	0.17	0.40	0.00	0.05	2.33	0.02	DNA
SctX	K04059	0.02	0.04	0.14	0.63	1.91	na	DNA
SctC	K03219	0.21	0.34	0.02	0.14	1.62	na	RNA
SctF	K03221	0.04	0.03	0.71	0.84	1.30	na	RNA
SctJ	K03222	0.28	0.79	0.01	0.09	2.78	na	RNA
SctL	K03223	0.04	0.07	0.37	0.56	1.91	na	RNA
SctN ATP synthase	K03224	0.73	1.99	0.00	0.07	2.71	na	RNA
SctR	K03226	0.18	0.62	0.00	0.04	3.41	0.03	RNA
SctS	K03227	0.08	0.24	0.01	0.07	3.03	na	RNA
SctT	K03228	0.04	0.15	0.01	0.12	3.38	na	RNA
SctU	K03229	0.23	0.38	0.07	0.23	1.63	na	RNA
SctV	K03230	0.98	2.42	0.00	0.03	2.47	na	RNA
SctW	K04058	0.03	0.04	0.56	0.73	1.24	na	RNA

*na* not applicable

**Table 3:** Gene set enrichment analysis of the metagenome dataset

KEGG pathway	NES	NOM *P*-val	FDR q-val	FWER *P*-val
ko03070-bacterial secretion system	1.70	0.019	0.470	0.386
ko00983-drug metabolism and other enzymes	1.55	0.039	0.688	0.773
ko00250-alanine, aspartate and glutamate metabolism	1.55	0.036	0.538	0.782

Pathways with genes differentially present or expressed based on nominal *P* value (NOM *P* ≤ 0.05) are shown, ranked by normalised enrichment score (NES). Corrected *P* values for false discovery rate (FDR q-val) and familywise-error rate (FWER *P* val) are shown.

### Bacterial T3SS genes in the rumen are associated with unidentified Proteobacteria and the genus *Succinivibrio*

**Fig. 3:**
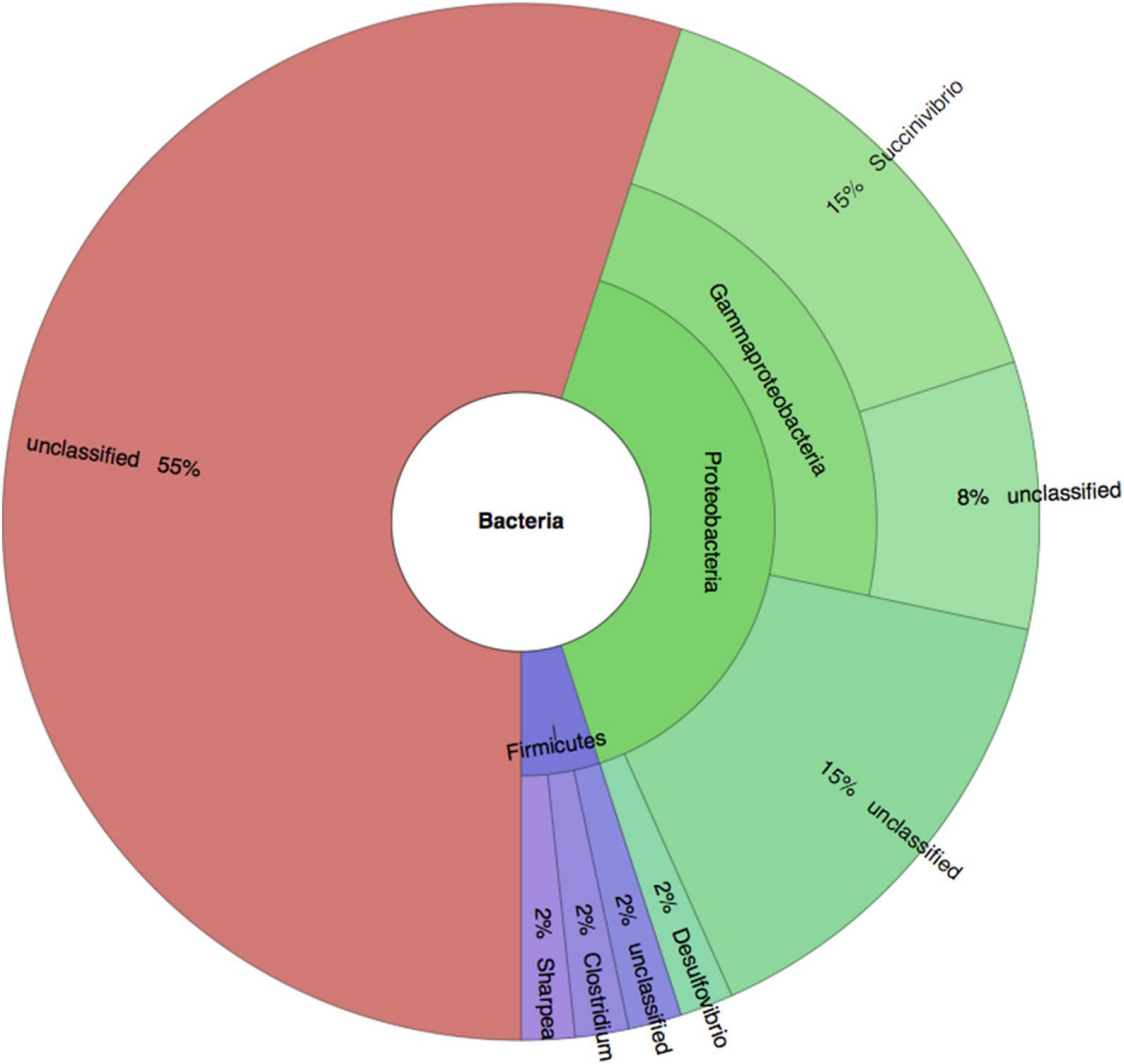
Phylogenetic affiliations of T3SS genes. *Sunburst plot* showing phylogenetic affiliations of contigs containing T3SS genes within the domain bacteria

To identity the origin of the T3SS genes found in the rumen metagenome and metatranscriptome sequences, we screened for contigs containing these genes, and a total of 101 genes were found on the 73 contigs retrieved. Using the lowest common ancestor algorithm in MEGAN, 82% of contigs were assigned to Bacteria, while the remaining 18% could not be assigned to any phylogenetic grouping. Some Bacteria-associated contigs could be further assigned (Fig. 3), with the largest group falling into the Proteobacteria (40%, 24 contigs). The largest genus-level group within the Proteobacteria was *Succinivibrio* (9 contigs, 12%). The phylogenetic affiliations of the *Succinivibrio* contigs were explored using manual BLAST searches against the IMG database. T3SS genes were identified in draft genomes of *Succinivibrio dextrinosolvens* strains H5, 22B, ACV-10, and DSM 3072 (Additional file 2). Further searches for T3SS KOs in bacteria isolated from the rumen showed that T3SS genes were also present in *Succinimonas amylolytica* DSM 2873 (type III secretion genes *yscC, J, L, N, R, S, T, U*, and *V*) and *Desulfotomaculum ruminis* DSM 2154 (*yscN*, ATP synthase type III secretion protein N [EC:3.6.3.14]) but in no other rumen bacteria.

**Fig. 4:**
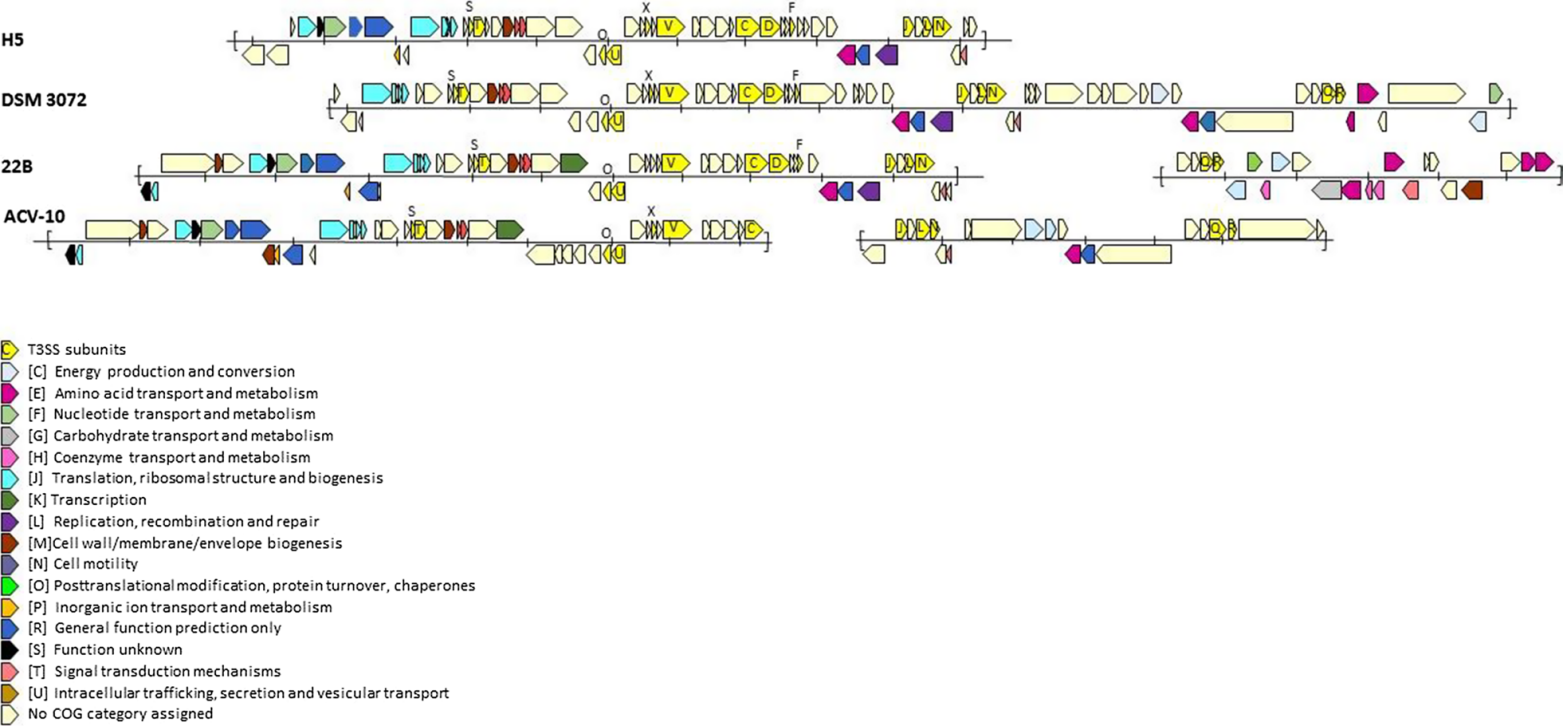
T3SS gene arrangement in genomes of *Succinivibrio dextrinosolvens* strains. *Sct* genes are shown in *yellow* with their associated gene subunit letter. Scaffold boundaries are shown by *square brackets*. The remaining genes are identified by COG categories

Analysis of the scaffold sequences of the draft genomes of *S. dextrinosolvens* strains H5, 22B, ACV-10, and DSM 3072, showed that the genes encoding the T3SS structural subunits are arranged in conserved structures (Fig. 4) with several other genes known to be associated with T3SSs. These include genes encoding low calcium response chaperones (LcrH/SycD), several Tir chaperone proteins (CesT), secretion system effector C-like family protein (SseC; involved in translocon formation) and TyeA proteins (involved in translocation of *Yersinia* outer proteins into eukaryotic cells). Analysis of the amino acid sequences of the predicted protein coding sequences of *S. dextrinosolvens* strains H5, 22B and ACV-10 using the EffectiveDB suite of software, identified proteins predicted to be secreted by T3SSs, proteins containing conserved chaperone binding domains in their N-terminal regions, eukaryotic-like N-terminal signal sequences, and also uncovered several T4SS effector proteins in each of the strains (summarised in Table 4, individual proteins are shown in Additional file 3).

**Table 4:** Effective T3 predictions of chaperone binding, secreted, N-terminal signal and T4SS proteins with protein coding sequences of *Succinivibrio dextrinosolvens*

Strain	Conserved chaperone binding (within/outside expected region)	Secreted T3SS	N-terminal signal (ER/mitochondrial)	T4SS effector
H5	111 (40/71)	254	447 (412/35)	126
22B	115 (36/79)	288	500 (452/48)	164
ACV-10	111 (35/76)	282	534 (493/41)	148

### *Succinivibrio dextrinosolvens* stimulates methane formation in the Methanomassiliicoccales affiliated methanogenic archaeon ISO4-H5

**Fig. 5:**
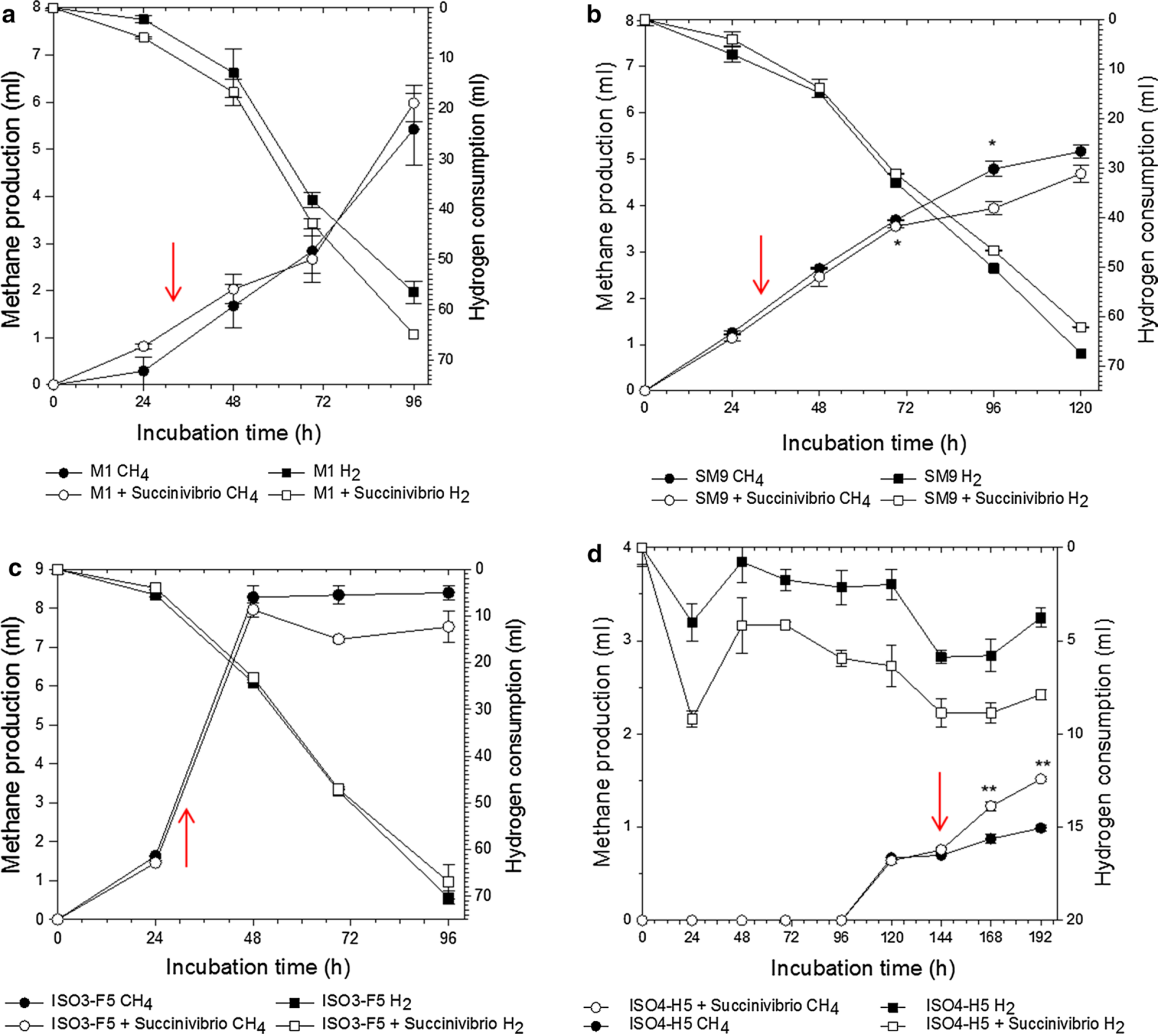
Co-cultures of rumen methanogens with *Succinivibrio dextrinosolvens* H5. Methane production and hydrogen consumption during co-culture experiments of *Succinivibrio dextrinosolvens* H5 with the rumen methanogens *Methanobrevibacter ruminantium* M1 (**a**), *Methanobrevibacter* sp. SM9 (**b**), *Methanosphaera* sp. ISO3-F5(**c**) and methanogenic archaeon ISO4-H5 (**d**). *Red arrows* indicate the time point of inoculation with *S. dextrinosolvens* H5

The preceding analyses indicated that T3SSs, some of which were similar to those found in ruminal strains of *Succinivibrio* spp., were more abundant within the microbiomes of HMY versus LMY animals, and indicate a possible association of *Succinivibrio* with the HMY phenotype. Therefore experiments were performed to explore the effects of *S. dextrinosolvens* strain H5 on methane formation by common hydrogenotrophic and methylotrophic methanogens from the rumen. Inoculation of *S. dextrinosolvens* H5 into methanogen cultures stimulated methane formation in the methanogenic archaeon sp. ISO4-H5 (Fig. 5). The stimulation of methane formation was observed 24 h after *S*. *dextrinosolvens* inoculation and became significant after 48 h of co-culture. *S. dextrinosolvens* inoculation had a slight inhibitory effect on *Mbb. millerae* SM9, reducing methane by 21% at 96 h relative to the control (*P* < 0.05). *S. dextrinosolvens* inoculation had no effect on methane formation in either *Mbb. ruminantium* M1 or *Methanosphaera* sp. ISO3-F5 (Fig. 5). Formate was the main VFA produced in the co-cultures (Table 5).

**Table 5:** Concentrations (mM) of volatile fatty acids in co-cultures

Culture	VFAs^a^										
	Formic		Acetic			Butryric				Propionic	
	Before/after	Net	Before/after		Net	Before/after		Net		Before/after	Net
ISO3-F5	0.31/0.00	−0.31	31.09/30.36		−0.73	0.84/0.83		−0.02		2.84/2.24	−0.59
ISO3-F5 + H5	0.06/1.13	1.07	31.92/32.48		0.56	0.82/0.86		0.04		2.41/2.30	−0.11
ISO4-H5	0.00/0.00	0.00	31.86/32.79		0.93	0.88/0.89		0.01		3.15/2.25	−0.91
ISO4-H5 + H5	0.00/1.88	1.88	32.87/34.62		1.75	0.87/0.87		0.01		2.51/2.70	0.19
M1	0.00/0.00	0.00	40.00/38.06		−1.95	0.78/0.68		−0.11		2.50/2.50	−0.01
M1 + H5	0.00/0.00	0.00	37.33/36.37		−0.97	0.84/0.82		−0.02		2.68/2.68	0.01
SM9	0.00/0.00	0.00	30.83/33.00		2.17	0.74/0.81		0.06		1.55/2.41	0.86
SM9 + H5	0.00/0.84	0.84	31.78/34.07		2.29	0.83/0.81		−0.01		2.52/2.30	−0.22

^a^No lactic acid was detected in any of the cultures. Negative numbers in the Net VFA columns indicates disappearance of that VFA during co-culture growth

## Discussion

The reduction of methane emissions from ruminants is being addressed via several lines of research, including the selection of animals based on their methane yields [8]. As the methane emission trait is heritable in sheep, there is scope to select for flocks with low methane emissions, and such breeding programmes need to understand the mechanisms underlying the trait to enhance the selection process and to avoid negative impacts on digestive processes and animal productivity. We have shown previously that the microbiome of LMY sheep has small differences in methanogen communities [19] but large differences in the expression of genes encoding the methanogenesis pathway [20]. We have also recently shown that the bacterial community in LMY animals is enriched for *Sharpea* spp. and expresses metabolic pathways that lead to lower hydrogen production and therefore lower methane formation. Here, the microbiomes of HMY animals have been analysed and we report the unusual finding that gene and transcript abundances of bacterial T3SS genes are positively correlated with HMY in sheep.

**Fig. 6:**
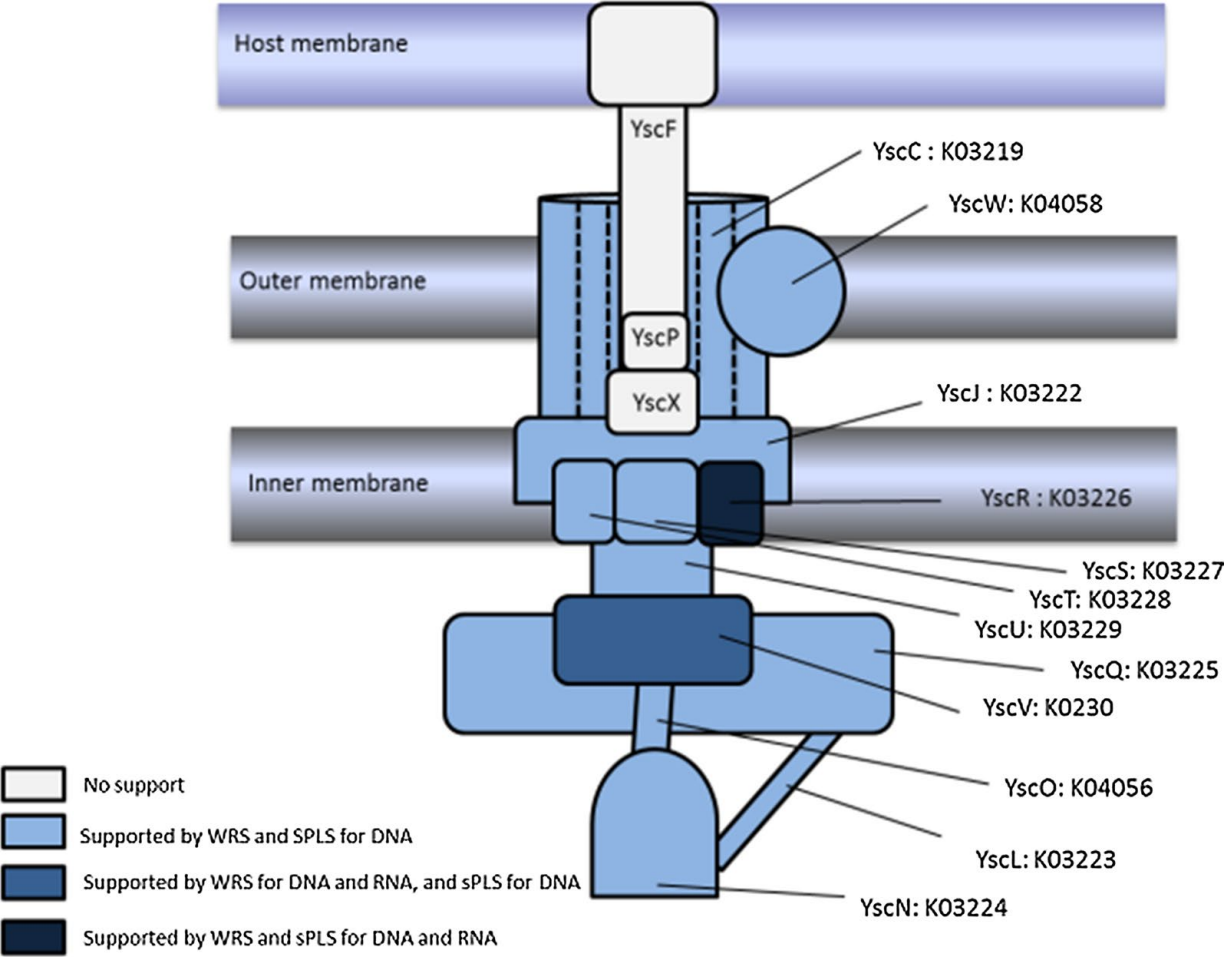
Schematic overview of T3SS subunits based on KEGG genes. The T3SS subunits are labelled using the *Yersinia* gene annotation scheme. The colour shading of each subunit shows the level of support for the related genes (DNA) or transcripts (RNA) being significantly (*P* ≤ 0.05) more abundant in HMY animals based on the WRS test and sPLS

The enrichment of T3SS genes in rumen metagenome sequences from HMY animals is surprising as T3SSs are uncommon in the rumen microorganisms that have been characterised to date. T3SS are most commonly found in pathogenic, Gram-negative bacteria where they are known as “injectisomes”, because they encode a protein complex that forms a needle-like appendage [39] which is used during infection to translocate effector proteins into the host to promote pathogen survival and resistance to the host immune system. The protein subunits which are typically found in T3SSs are the cytoplasmic subunits (SctQ, L, O, and N which make up the cytoplasmic ring protein, stator, stalk, accessory protein, and ATPase respectively); the export apparatus (SctV, U, R, S, and T which are the major export protein, switch protein, three minor export proteins, respectively) and the base, and needle proteins (SctJ, D, I, C, P, X and F which are the inner Membrane Supramembrane (MS) ring, the outer MS ring, the inner rod, the secretin, the needle length regulator, a secretion protein and the needle filament, respectively). Representatives of nearly all the genes encoding subunits required for T3SS assembly were identified in the metagenome of HMY animals, including the cytoplasmic components (SctQLON), the export proteins (SctVURST) and the base and needle proteins (SctJDICF) and their occurrence and level of statistical support are shown in Fig. 6. Interestingly, genes encoding the needle protein (SctF), the needle length regulator (SctP) and the putative animal-specific secretion protein (SctX) were present at very low levels and were not enriched in HMY animals. T3SS in human pathogenic bacteria are also known to allow invasion of other hosts, including free-living amoebae and protozoa, which forms important environmental reservoirs for these pathogens and may protect the internalized bacteria from detection and treatment with biocides [40]. Although most common in pathogens, T3SS are also found in bacteria that engage in symbiotic relationships with their animal or plant hosts, and in this context the role of T3SS is thought to be in determining the specificity and maintenance of the symbiotic interaction [41].

The majority of T3SS genes found in the metagenome dataset could not be assigned a particular phylogenetic origin, and appear to belong to unidentified and/or uncultured members of the rumen microbiome. Of those sequences that could be assigned, most were affiliated with the phylum Proteobacteria, and at the genus level to *Succinivibrio*, which includes as its only species the succinate-producing *S. dextrinosolvens* [42]. Overall, the occurrence of genes encoding T3SSs in rumen bacteria was low; using available genome sequences, T3SS genes were only found in four strains of *Succinivibrio dextrinosolvens*, in another succinate-forming bacterium *Succinimonas amylolytica* DSM 2873 [43] and one gene (*yscN*) was found in the sulphate-reducing bacterium *Desulfotomaculum ruminis* DSM 2154 [44]. None of the ruminal strains of *Sharpea*, or the human gut organism *Akkermansia mucinophila* (the only genome available from a member of the phylum Verrucomicrobia isolated from a gut environment), contained T3SS gene homologues, therefore it is likely that the T3SS assigned to these genera by MEGAN belong to unidentified ruminal species.

From examination of their draft genome sequences, strains of the rumen bacterial species *S. dextrinosolvens* appear to encode complete T3SS, and along with *Succinimonas amylolytica* are the only two characterised rumen bacteria that have this type of secretory system. *S. dextrinosolvens* is a Gram negative, curved rod-shaped bacterium that degrades starch, and produces succinate, acetate, formate and sometimes lactate as its main end products of fermentation [42]. It is known to be part of the epimural (adherent to the rumen wall) community in the bovine [45, 46] and is typically enriched when the diet contains high levels of starch [47, 48]. *Succinivibrio* are considered to be a small, but consistent part of the normal rumen microbiome [49] and is not known to be pathogenic to ruminants, although it has been reported as the infective agent in 2 cases of human disease [50, 51]. It seems unlikely that *Succinivibrio* spp. use T3SS for mounting infections, and it is more plausible that it mediates non-lethal relationships, either with other rumen organisms, or possibly the animal host itself. In pathogenic bacteria, effector proteins secreted by the T3SS act to modulate host cell functions to help avoid immune detection and disable protective functions such as macrophages [52]. An amino acid sequence-based analysis of the protein-coding genes of *S. dextrinosolvens* strains H5, 22B and ACV-10 identified a large number of proteins containing N-terminal motifs indicative of involvement in T3SSs (Table 4), including chaperone binding domains and eukaryotic-like signal sequences specific for the endoplasmic reticulum or mitochondria (Additional file 2). The annotated functions of these proteins were quite diverse but many of them have putative roles in transcriptional regulation or transport functions, indicating they may mediate changes in gene expression and transport of molecules in the target organisms.

The specific contribution of the T3SS genes detected in the microbiomes of the HMY animals to the HMY phenotype remains unclear. The HMY phenotype in sheep is thought to be related to rumen size, particle retention time and turnover rate, such that HMY animals have a larger rumen which retains feed particles for longer and therefore have a slower turnover rate. These conditions are thought to lead to low hydrogen partial pressure, but rapid hydrogen production, which results in elevated expression of genes encoding the hydrogenotrophic methanogenesis pathway [20]. The assignment of several of the T3SS genes to *Succinivibrio* suggests that this bacterial genus may be important in the HMY microbiome. However, this does not appear to be the case as the 16S rRNA genes retrieved from the metagenome sequences or from amplicon sequencing did not show *Succinivibrio* spp. as being significantly enriched in either the LMY (0.065% relative abundance) or HMY animals (0.033%). Investigations of the microbiomes of cattle with differing methane yield, have shown that OTUs assigned at a higher family level to the Succinivibrionaceae were more abundant in the microbiomes of LMY animals [53], while another study of cattle has reported OTUs corresponding to Succinivibrionaceae were reduced in feed restricted animals compared to ad libitum fed animals [54]. The majority of T3SS genes which make up the main component of the correlation with methane yield to appear to come from unidentified rumen bacteria, but without knowledge of their metabolisms or physiologies, it is not possible to predict how their T3SS may influence the methane yield phenotype.

The co-culture experiments showed that *S*. *dextrinosolvens* had either stimulatory, inhibitory or neutral effects, depending on the type of methanogens in the co-culture. *Succinivibrio* spp. have been observed on several occasions in enrichment cultures with methanogens of the order Methanomassiliicoccales, and have proven difficult to remove from these enrichments to allow purification of the methanogens [55]. This suggests that *Succinivibrio* spp. form close relationships with Methanomassiliicoccales, and helps explain the stimulation of ISO4-H5 by *S. dextrinosolvens* H5 in co-cultures. In a global census of microbes from ruminant animals, Henderson et al. [49] reported a positive association between succinate-producing Succinivibrionaceae, and methanogens belonging to the family Methanomassiliicoccaceae, particularly the subgroups, *Candidatus* Methanomethylophilus alvus and Methanomassiliicoccales group 11 sp. The mechanism of stimulation is currently not known, but it is possible that *Succinivibrio* spp. provide substrates or growth factors that stimulate the growth and methane formation in the methanogenic archaeon ISO4-H5. Some *Succinivibrio* spp. are able to degrade pectin [42] and release methanol [56] which is a known substrate for methane formation by the Methanomassiliicoccaceae [57].

The inhibition of methane formation during *S. dextrinosolvens* co-culture with *Mbb. millerae* SM9 is interesting as the Succinivibrionaceae bacterium WG-1 isolate from the Wallaby gut has been implicated in lower methane emissions from starch-containing diets, presumably via close coupling of redox reactions which led to less methane being formed [58]. The global census of rumen microbes also reported a negative association between Succinivibrionaceae and the *Methanobrevibacter gottschalkii* clade [49]. In our co-cultures, hydrogen was provided in excess, so the weak inhibition of *Methanobrevibacter* sp. SM9 by *S. dextrinosolvens* may be via a direct effect, rather than via competition for hydrogen. This is possibly via the production of formate by *S. dextrinosolvens* in the co-culture, which cannot be used for methane formation by *Mbb. millerae* [59] and which can slow growth when added to *Mbb. millerae* SM9 cultures growing on H_2_ + CO_2_ (Peter Janssen, pers. comm.). Investigating the exact mechanisms by which *Succinivibrio* spp. influences methanogen growth and methane formation, and the potential involvement of their T3SSs, are the subject of on-going research.

## Conclusions

Bacterial T3SS genes and transcripts, were found to be positively correlated with methane yield in sheep. Most of these genes could not be assigned phylogenetically, but several were affiliated with the genus *Succinivibrio,* and genes encoding complete T3SSs were found in the genome sequences of *S. dextrinosolvens* strains H5, 22B and ACV-10, and another rumen succinate-producing rumen bacterium, *Succinimonas amylolytica*. This is the first report of T3SS genes being associated with methane emissions in ruminants, and identifies these secretions systems as potential new targets for methane mitigation research. *S*. *dextrinosolvens* H5 was shown to have direct growth-enhancing effects on a member of the Methanomassiliicoccales, and an inhibitory effect on a member of the *Mbb. gottschalkii* clade in co-culture experiments, which point towards bacteria-methanogen interactions being important modulators of methane production in ruminant animals.

## Additional files

**Additional file 1.** KEGG genes significantly more abundant in HMY animals in the metagenome and metatranscriptome datasets by WRS and sPLS analyses.

**Additional file 2.** Type III secretion system Kos in foregut bacteria in the Integrated Microbial Genome.

**Additional file 3.** Effective analysis of protein coding sequences in *S. dextrinosolvens* strains H5, 22B and ACV-10.

## Abbreviations

HMY: high methane yield
LMY: low methane yield
DMI: dry matter intake
IMY: intermediate methane yield
GSE: gene set enrichment analysis
sPLS: sparse partial least squares
WRS: Wilcoxon Rank Sum
T3SS: type III secretion system
KEGG: Kyoto encyclopedia of genes and genomes
MSPE: mean squared prediction error
NES: normalised enrichment score
IMG/Mer: integrated microbial genomes and microbiomes (expert review)
FDR: false discovery rates
BH: Benjamini–Hochberg procedure
MEGAN: metagenome analyser
MS: membrane supramembrane
RPM: reads per million
NOM *P*-val: nominal *P* value
FWER: familywise-error rate
